# Incremental prognostic value of lung ultrasound on contemporary heart failure risk scores

**DOI:** 10.3389/fphys.2022.1006589

**Published:** 2022-09-14

**Authors:** Alba Maestro-Benedicto, Mercedes Rivas-Lasarte, Juan Fernández-Martínez, Laura López-López, Eduard Solé-González, Vicens Brossa, Sonia Mirabet, Eulàlia Roig, Juan Cinca, Jesús Álvarez-García, Alessandro Sionis

**Affiliations:** ^1^ Cardiology Department, IIB SANT PAU, Hospital de la Santa Creu i Sant Pau CIBERCV, Barcelona, Spain; ^2^ Cardiology Department, Hospital Universitario Puerta de Hierro CIBERCV, Majadahonda Madrid, Spain; ^3^ Cardiology Department, Hospital Clinic, Barcelona, Spain; ^4^ Cardiology Department, Hospital Universitario Ramón y Cajal CIBERCV, Madrid, Spain

**Keywords:** congestion, heart failure, lung ultrasound, scores, biomarkers, prognosis

## Abstract

**Introduction:** Over the last decades, several scores have been developed to aid clinicians in assessing prognosis in patients with heart failure (HF) based on clinical data, medications and, ultimately, biomarkers. Lung ultrasound (LUS) has emerged as a promising prognostic tool for patients when assessed at discharge after a HF hospitalization. We hypothesized that contemporary HF risk scores can be improved upon by the inclusion of the number of B-lines detected by LUS at discharge to predict death, urgent visit, or HF readmission at 6- month follow-up.

**Methods:** We evaluated the discrimination improvement of adding the number of B-lines to 4 contemporary HF risk scores (Get with the Guidelines -GWTG-, MAGGIC, Redin-SCORE, and BCN Bio-HF) by comparing the change in the area under the receiver operating curve (AUC), the net reclassification index (NRI), and the integrated discrimination improvement (IDI). The population of the study was constituted by the 123 patients enrolled in the LUS-HF trial, adjusting the analyses by the intervention.

**Results:** The AUC of the GWTG score increased from 0.682 to 0.789 (*p* = 0.02), resulting in a NRI of 0.608 and an IDI of 0.136 (*p* < 0.05). Similar results were observed when adding the number of B-lines to the MAGGIC score, with an AUC that increased from 0.705 to 0.787 (*p* < 0.05). This increase translated into a NRI of 0.608 and an IDI of 0.038 (*p* < 0.05). Regarding Redin-SCORE at 1-month and 1-year, the AUC increased from 0.714 to 0.773 and from 0.681 to 0.757, although it did not reach statistical significance (*p* = 0.08 and *p* = 0.06 respectively). Both IDI and NRI were significantly improved (0.093 and 0.509 in the 1-month score, *p* < 0.05; 0.056 and 0.111 in the 1-year score, *p* < 0.05). Lastly, the AUC for the BCN Bio-HF score increased from 0.733 to 0.772, which was statistically non-significant, with a NRI value of 0.363 (*p* = 0.06) and an IDI of 0.092 (*p* < 0.05).

**Conclusion:** Adding the results of LUS evaluated at discharge improved the predictive value of most of the contemporary HF risk scores. As it is a simple, fast, and non-invasive test it may be recommended to assess prognosis at discharge in HF patients.

## 1 Introduction

Risk prediction in heart failure (HF) remains essential to identify those patients who may benefit from a closer management. Since the turn of the century, several scores have been proposed and externally validated (Levy et al., 2006; Peterson et al., 2010; Pocock et al., 2013; Lupón et al., 2014; Álvarez-García et al., 2015). Most can be easily calculated using demographics, laboratory, and medication data, and are available through free-access websites. Nevertheless, no predictive scale has been found uncontrovertibly better than the rest (Codina et al., 2021), illustrating the complexity of risk prediction in HF.

As HF treatment has dramatically evolved during the last decades, existing prognostic scores, have been continuously updated adding emerging data from both newer therapeutic and diagnostic tools (Sinha et al., 2021). Particularly, lung ultrasound (LUS) has emerged in the last years as a simple and non-invasive instrument for detecting pulmonary congestion in patients with HF. Its prognostic value has been assessed in different clinical scenarios (Coiro et al., 2015; Coiro et al., 2015; Gargani et al., 2015; Platz et al., 2016; Scali et al., 2017; Coiro et al., 2020; Rivas-Lasarte et al., 2020; Domingo et al., 2021; Gargani et al., 2021; Kobayashi et al., 2021; Mazzola et al., 2021; Pugliese et al., 2021; Pugliese et al., 2021), showing that the presence of B-lines detected by LUS is associated with an increased risk of worse outcomes.

Thus, we hypothesized that contemporary risk scores can be improved by incorporating the number of B-lines detected by LUS at HF discharge to predict death or hospital readmission at 6- month follow-up.

## 2 Material and methods

### 2.1 Study design

This is a sub-analysis including 123 patients enrolled in the LUS-HF trial, whose study design and primary results have been previously reported (Rivas-Lasarte et al., 2019). In brief, the LUS-HF was a single-center, single-blind, randomized clinical trial evaluating tailored LUS-guided diuretic treatment of pulmonary congestion in patients with HF. Patients were required to be aged ≥18 years and to have been hospitalized for HF defined by shortness of breath, pulmonary congestion on X-ray, and elevated N-terminal pro B-type natriuretic peptide (NT-proBNP) values in the first 24 h of admission (cut-off values: 450 ng/L in patients aged <50 years; >900 ng/L in patients aged 50–75 years; >1800 ng/L in patients aged >75 years). Exclusion criteria included inability to attend follow-up visits, life expectancy of <6 months, haemodialysis, and the presence of severe lung disease preventing LUS interpretation. Eligible patients were randomized at discharge to either the non-LUS-guided group (control group) or the LUS-guided group (LUS group). Visits were scheduled in the HF clinic at 14, 30, 90, and 180 days after discharge. LUS was performed in both groups, but the result was only available to the treating physician in the LUS-guided arm.

The primary endpoint was a composite of urgent visit, hospitalization for worsening HF, and death at 6 months. Urgent visits for worsening HF were defined as visits to the emergency department or un-scheduled visits to the HF unit as a result of signs and/or symptoms of worsening HF that required intravenous diuretic treatment or diuretic increase with a hospital stay of <24 h. Hospitalization for worsening HF was defined as a stay in hospital for >24 h mainly as a result of signs and/or symptoms of worsening HF. The reported events were reviewed by an independent panel of investigators.

The protocol was approved by the ethics committee and the study was conducted in accordance with the principles of the Declaration of Helsinki. Written informed consent was obtained from all patients prior to study participation.

### 2.2 Lung ultrasound protocol

According to current expert recommendations (Platz et al., 2019), LUS was recorded using a pocket ultrasound device (VScan, General Electrics) with a cardiac phased array transducer at four sites in each hemithorax (mid clavicular, mid axillar superior and inferior in each side) with the transducer perpendicular to the ribs and at a 16 cm imaging depth being the patient in the semi-recumbent position. The number of B-lines reported was the sum of the B-lines visualized in each thoracic site.

For this post-hoc analysis, the number of B-lines detected by LUS at discharge was analysed.

### 2.3 Contemporary HF risk scores

We selected 4 contemporary scores: Get with the Guidelines, MAGGIC, Redin-SCORE and BCN Bio-HF.

#### 2.3.1 The get with the guidelines–heart failure score (GWTG- HF) (Peterson et al., 2010)

The GWTG-HF score incorporates 9 variables (age, systolic blood pressure (BP), body mass index (BMI), total cholesterol, high-density lipoprotein cholesterol, QRS duration, smoking status, use of antihypertensive medication, use of diabetes medication) to predict the risk of in-hospital mortality for patients hospitalized with HF.

#### 2.3.2 The meta-analysis global group in chronic heart failure score (MAGGIC) (Pocock et al., 2013)

The MAGGIC score was derived from a metanalysis of 30 studies to predict mortality rates in patients with HF, and includes 13 predictors: age, lower ejection fraction (EF), New York Heart Association (NYHA) class, serum creatinine, diabetes, not prescribed beta-blocker, lower systolic BP, lower BMI, time since diagnosis, current smoker, chronic obstructive pulmonary disease, male gender, and not prescribed angiotensin converter enzyme inhibitors (ACEi) or angiotensin-receptor blockers.

#### 2.3.3 The Redin-SCORE (Álvarez-García et al., 2015)

The Redin-SCORE is a risk score developed to predict short-term (1 month) and long-term (1 year) risk of HF in ambulatory patients. Predictors of 1-month readmission were the presence of elevated natriuretic peptides, left ventricular (LV) HF signs, and estimated glomerular filtration rate (eGFR) < 60 ml/min/m2. Predictors of 1-year readmission were elevated natriuretic peptides, anaemia, left atrial size >26 mm/m2, heart rate >70 beats per minute (bpm), LV HF signs, and eGFR <60 ml/min/m2.

#### 2.3.4 The BCN Bio-HF score (Lupón et al., 2014)

The first version of the BCN Bio-HF included clinical variables, medications, conventional laboratory analytes (sodium, estimated glomerular filtration rate), and NT-proBNP, high-sensitivity troponin T (hs-TnT), and interleukin-1 receptor-like-1 (known as ST2). It was updated in 2018 by incorporating the use of angiotensin receptor neprylisin inhibitor (ARNI), cardiac resynchronization therapy (CRT), and implantable cardioverter defibrillator (ICD).

### 2.4 Statistical analysis

Continuous variables are expressed as mean (standard deviation) or as median (interquartile range) whenever appropriate. Differences in continuous variables were tested by the analysis of variance (ANOVA), Student’s t-test, or Wilcoxon signed rank test for independent samples. Categorical variables were presented as frequency and percentage. Differences in the categorical variables were assessed by the χ2 test or by Fisher’s exact test.

Discrimination, calibration and reclassification methods are recommended when evaluating candidate variables in prognostic studies (Januzzi et al., 2014). Thus, we first assessed the discriminative ability of each selected HF score to predict the occurrence of the primary endpoint at 6 months in the study population by calculating the area under the receiver operating curve (AUC). Thereafter, we analysed the incremental prognostic value of the number of B-lines at discharge on top of each score by comparing the AUC with and without LUS data and calculated the integrated discrimination improvement (IDI), and net reclassification improvement (NRI). Finally, we also performed decision curve analysis (DCA) to visualize the net benefit for clinical decisions. Data were analysed using STATA SE Version 15.0 (StataCorp LLC, College Station, TX, United States). A two-sided *p* < 0.05 was considered significant.

## 3 Results

### 3.1 Characteristics of the study population and number of B-lines at discharge

Clinical characteristics of the LUS-HF population and LUS data at discharge are shown in Table 1. Briefly, median age of the patients was 70 years, most patients were male (72%), had a reduced LVEF (55%), and a high prevalence of comorbidities. Median number of B-lines at discharge was 4 (Levy et al., 2006; Pocock et al., 2013; Lupón et al., 2014; Álvarez-García et al., 2015; Codina et al., 2021; Sinha et al., 2021) and 41 patients (33%) had ≥5 B-lines.

**TABLE 1:** Baseline characteristics of the study population.

	Total (N = 123 patients)
Age, years	69 ± 12
Female sex	34 (28%)
BMI, kg/m2	26.8 ± 5.4
Cardiovascular risk factors	
Hypertension	89 (72%)
Dyslipidaemia	84 (68%)
Diabetes	50 (41%)
Smokers	25 (20%)
Comorbidities	
COPD	31 (25%)
Renal insufficiency*	46 (37%)
Stroke	19 (15%)
Anaemia**	25 (20%)
Charlson index	2.7 ± 1.6
Previous cardiac history	
Previous HF	68 (55%)
Ischemic HF aetiology	54 (44%)
Atrial fibrillation	68 (55%)
Median LVEF (%)	36 (30–49)
HFrEF	68 (55%)
HFmrEF	25 (21%)
HFpEF	28 (23%)
Characteristics at discharge	
Systolic blood pressure, mmHg	130 ± 24
Heart rate, b.p.m	68 ± 11
eGFR, mL/kg/min/1.73m2	63 ± 24
NT-proBNP, ng/L	1723 (884–3,776)
Peripheral oedema	21 (17%)
Pulmonary rales	23 (19%)
Treatment at discharge	
Loop diuretics	94 (76%)
Thiazide diuretics	4 (3%)
ACE inhibitors/ARB	75 (61%)
Sacubitril/valsartan	5 (4%)
Beta-blocker	104 (85%)
Mineralocorticoid receptor antagonist	36 (29%)
Implantable cardioverter-defibrillator	18 (15%)
Cardiac resynchronization therapy	7 (6%)
LUS data at discharge	
Number of B-lines	4 (2-7)
Pleural effusion	11 (9%)
Outcomes at 6 months	
Composite endpoint	39 (32%)
Heart failure admission	27 (22%)
Urgent visits for worsening HF	16 (13%)
Death	5 (4%)

*Renal insufficiency refers to eGFR <60 ml/min/1.73 m2.

**Anaemia refers to haemoglobin levels of <13 g/dl in men and <12 g/dl in women.

Data are expressed as number (%), mean ± standard deviation, or median (interquartile range), as appropriate.

ACE, angiotensin-converting enzyme; ARB, angiotensin-receptor blocker; BMI, body mass index; COPD, chronic obstructive pulmonary disease; eGFR, estimated glomerular filtration rate; HF, heart failure; HFpEF, heart failure with preserved ejection fraction; HFrEF, heart failure with reduced ejection fraction; LUS, lung ultrasound; LVEF, left ventricular ejection fraction.

### 3.2 Incremental prognostic value of LUS over contemporary heart failure risk scores

**FIGURE 1:**
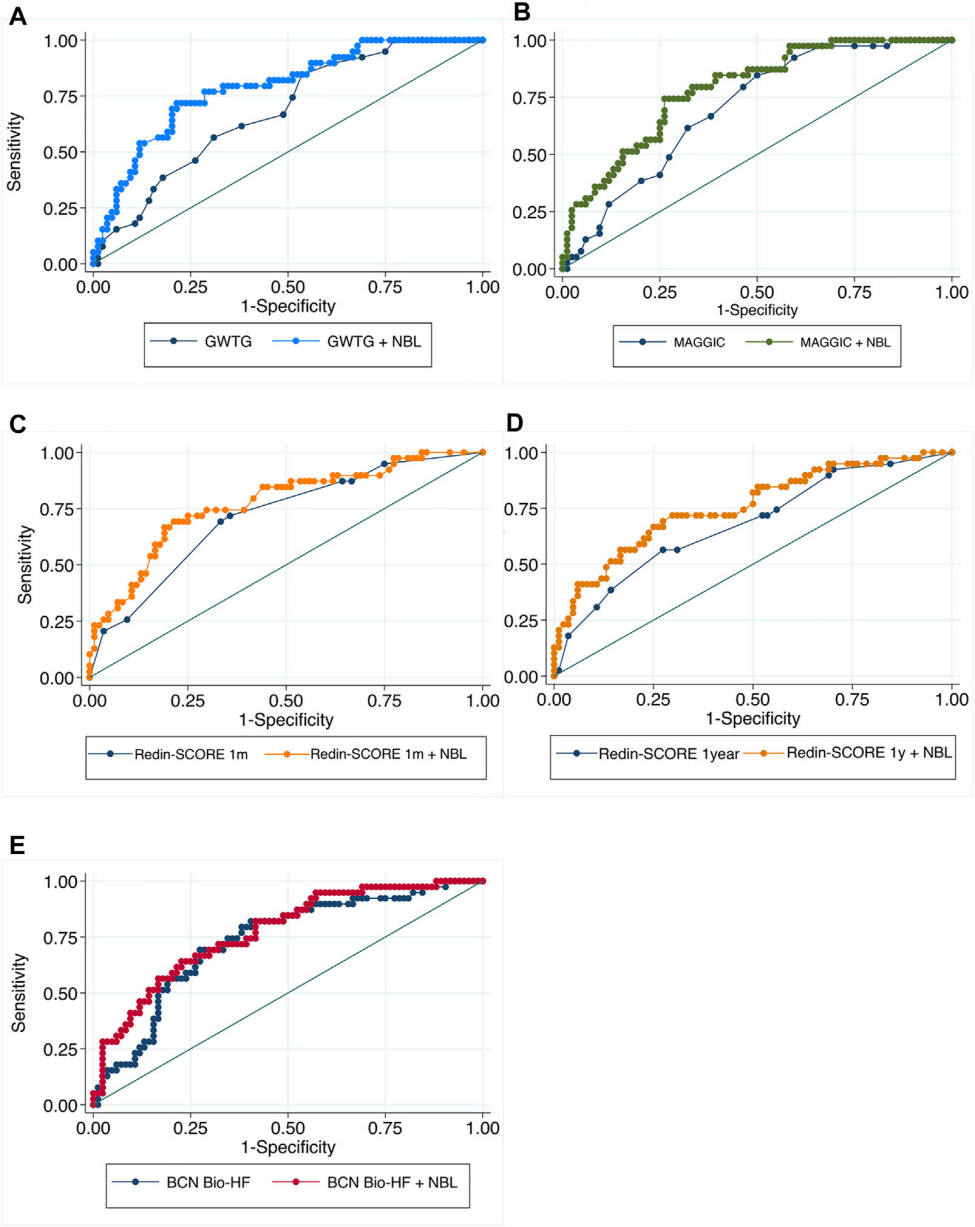
Comparison between the Receiver Operating Characteristic curves (ROC) for the composite endpoint at 6-month follow-up: score alone versus score + number of B-lines. ROC curves compare sensitivity versus specificity across a range of values for the ability of the score to predict the composite endpoint. Each patient is given a score with the intention that the test will be useful in predicting event occurrence and the different points on the curve correspond to the different cutpoints used to determine whether the test results are positive. Adding B-lines to GWTG, MAGGIC and REDIN-Score 1 year scores **(A,B,D)** makes the true positive rate higher and the false positive rate lower at all cutpoints compared with the score alone. Regarding BCN Bio-HF and REDIN-Score 1 m **(C,E)** adding B-lines improves both sensitivity and specificity in almost all cutpoints.

Table 2 summarizes the discrimination, calibration, IDI, and NRI parameters by the 4 HF scores for the primary outcome alone and in combination with the number of B-lines. Overall, the addition of the number of B-lines at discharge improved the AUC of each risk score (Figure 1). However, the incorporation of the number of B-lines only reached statistically significance for the GWTG and MAGGIC scores, but not for the Redin-SCORE at 1-month and 1-year, nor the BCN Bio-HF.

**TABLE 2:** Incremental prognostic value of B-lines to predict 6-month outcomes in the LUS-HF trial.

	AUC	*p* Value	AIC	BIC	H-L *p* value	IDI	NRI
GWTG score	0.682 (0.587–0.778)		148	153	0.3		
GWTG score **+** number of B-lines	0.798 (0.704–0.783)	0.018	131	136	0.2	0.136 (*p* < 0.001)	0.608 (*p* = 0.002)
MAGGIC score	0.705 (0.614–0.797)		146	152	0.5		
MAGGIC score **+** number of B-lines	0.787 (0.706–0.869)	0.045	131	137	0.3	0.119 (*p* < 0.001)	0.608 (*p* = 0.002)
BCN Bio-HF	0.733 (0.639–0.827)		145	150	0.2		
BCN Bio-HF **+** number of B-lines	0.772 (0.685–0.859)	0.340	133	139	0.4	0.092 (*p* = 0.003)	0.363 (*p* = 0.060)
Redin-SCORE 1-month	0.714 (0.621–0.808)		141	147	0.3		
Redin-SCORE 1-month **+** number of B-lines	0.773 (0.621–0.864)	0.08	130	135	0.2	0.093 (*p* = 0.003)	0.509 (*p* = 0.009)
Redin-SCORE 1-year	0.681 (0.579–0.783)		146	152	0.6		
Redin-SCORE 1-year **+** number of B-lines	0.757 (0.663–0.851)	0.056	133	137	0.9	0.056 (*p* = 0.004)	0.111 (*p* < 0.001)

AUC, area under the curve; AIC, akaike criteria; BIC, bayesian criteria; H-L, Hosmer-Lemeshow; IDI, integrated discrimination improvement index; NRI, net reclassification improvement index.

**FIGURE 2:**
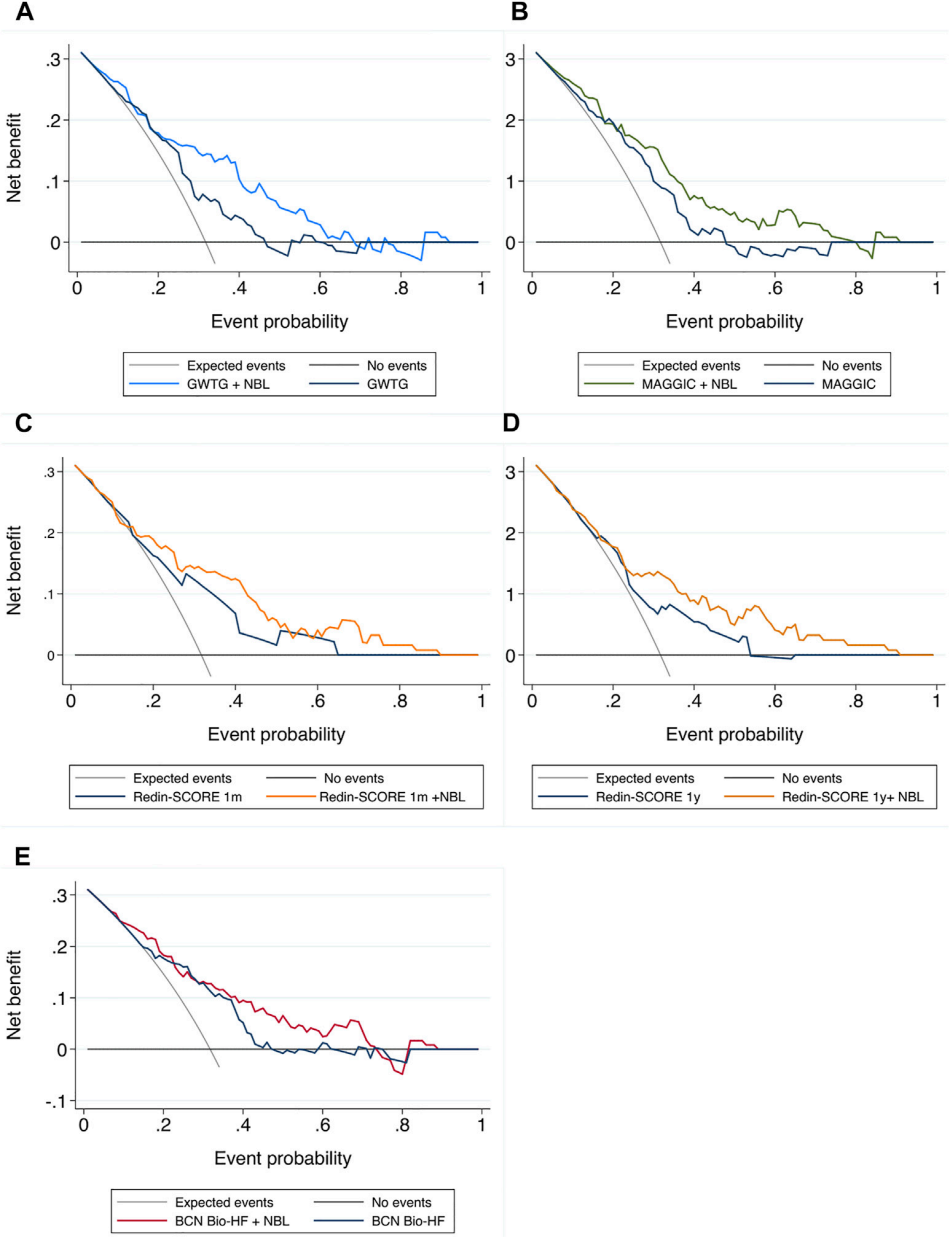
Calibration plots. X-axis: predicted outcome; Y-axis: observed outcome. NBL: number of B lines **(A)** GWTG: Get With the Guidelines score; **(B)** MAGGIC score; **(C)** Redin-SCORE 1 month; **(D)** Redin-SCORE 1 year; **(E)** BCN Bio-HF score.

**FIGURE 3:**
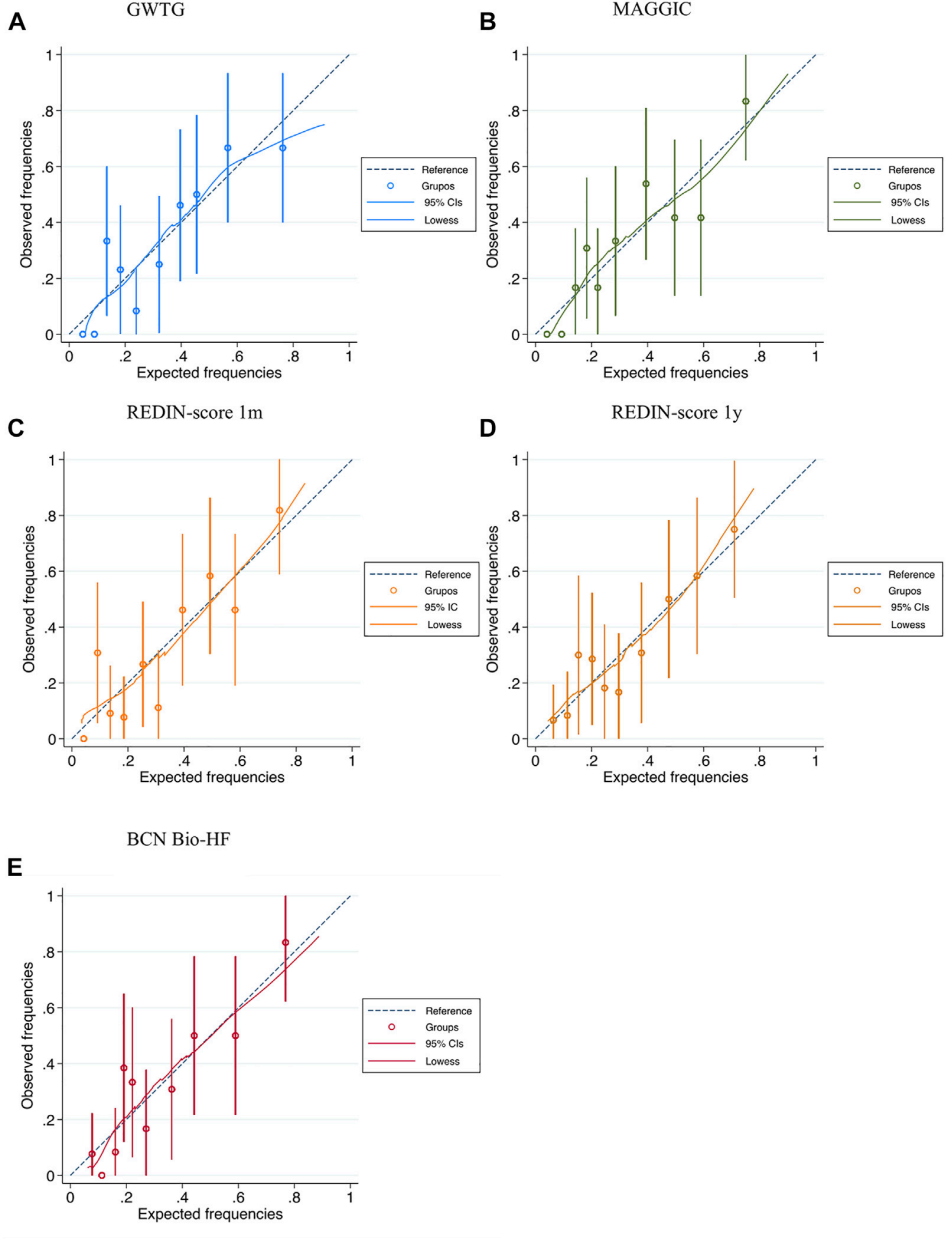
Decision curve analysis for predicting the primary composite endpoint. Decision curve analysis illustrates the performance of the model in a range of threshold probabilities, which may of interest to the clinician making the decision. X axis represents the probability threshold for the composite endpoint according to the score. The y axis represents the net benefit ([true positives - w x false positives]/total number of patients): positive values indicate an improvement in the classification of patients, and w is a correction factor for the probability threshold. The upper limit is 0.32 because the incidence of readmission for HF in LUS-HF was 32%. The diagonal black line assumes that all expected patients were readmitted, 32% at 6 months. The coloured lines represent the result of applying the different scores. Adding B-lines provided a net benefit due to better classification of the patients for probabilities below 70% in GWTG and BCN Bio-HF scores **(A,E)**. When B-lines were incorporated to MAGGIC score **(B)**, a net benefit was obtained due to better classification of the patients for probabilities between 0 and 80%. Regarding REDIN-score 1 year and 1m, a net benefit was obtained in all probability spectrum when using LUS data **(C,D)**.

Regarding reclassification indexes, both NRI and IDI after adding the number of B-lines showed a significant improvement with all scores, except for NRI in BCN Bio-HF score. As Figure 2 shows, the calibration curves indicating good concordance. Finally, Figure 3 displays the DCA, showing that the net benefit of adding LUS data was higher than that of the score alone for any threshold probabilities, except for the GWTG score, which applied only for an event probability under 70%.

## 4 Discussion

### 4.1 Main findings

Our work shows that the predictive value of contemporary HF risk scores can be improved by integrating LUS.

### 4.2 Prognostic value of LUS over HF risk scores

Prior research (Bettencourt et al., 2004; Khanam et al., 2018; Lupón et al., 2018) has already focused in analysing the prognostic value of new clinical variables to allow better prediction, such as the incorporation of ARNI or the effect of adding natriuretic peptides. The BCN Bio-HF score was one of the pioneers developing an updated version integrating those variables that allowed a better risk prediction.

LUS has emerged in the last decade as a simple, fast, and non-invasive test for lung congestion quantification. Several studies have shown that it might be a better tool for detecting subclinical pulmonary congestion than clinical assessment (Platz et al., 2016; Pellicori et al., 2019) and it has become widely available in an increasing number of centres, with the generalization of echographic equipments including pocket devices.

As NT-proBNP, it has also been reported that the presence of B-lines in HF patients is an independent prognostic factor (Coiro et al., 2015; Gargani et al., 2015; Gustafsson et al., 2015; Aras and Teerlink, 2016; Platz et al., 2016; Rivas-Lasarte et al., 2020; Domingo et al., 2021) although no study to date has analysed its prognostic value when added to the most used contemporary risk scores. To the best of our knowledge, this is the first study analysing if risk scores can be improved upon by the inclusion of B-lines detected by LUS at discharge and we found that the predictive yielding improved in a different degree according to the presence or absence of hemodynamic or biochemic markers of left ventricular function in their respective models.

Moreover, the number of B-lines is not only a prognostic marker but has shown to be also a therapeutic target in HF patients improving their prognosis when monitored during follow-up, mainly due to a reduction of HF decompensations (Rivas-Lasarte et al., 2019). As it is a dynamic marker that evolves with therapeutic measures, we hypothesize that its changes may also be of interest in predicting prognosis, although this remains to be elucidated in further studies.

### 4.3 Clinical implications

Risk stratification remains essential in HF to make medical decisions based on life expectancy and develop appropriate treatment plans, but the accuracy of available prognostic risk scores in patients with HF is still limited. Our study contributes on this important issue by integrating in existing contemporary HF risk scores LUS and allowing for a significant improvement in their predictive value in the majority of cases.

Some variables included in the pre-existing predictive HF models are not frequently obtained in the clinical care of HF, but LUS can be performed quickly and easily at bedside, and has already become an add-on to lung auscultation for the evaluation of pulmonary congestion.

As a semi-quantitative measure of pulmonary congestion, LUS adds new and valuable information to the scores. Due to its dynamic behaviour, it can be used as a monitoring tool allowing reassessment of patient’s status whenever clinical situation changes, and also as a therapeutic target. Several studies had proved a LUS-guided therapy reduces acute decompensation events in the follow-up (Rivas-Lasarte et al., 2019; Araiza-Garaygordobil et al., 2020; Marini et al., 2020; Mhanna et al., 2021; Rastogi et al., 2022), which explains its rapid and wide implementation in the HF field.

### 4.4 Study limitations

Our study has some limitations. First, we tested prognostic scores which were specifically designed for ambulatory HF patients in a sample that was comprised by HF patients discharged from hospital. Second, LUS-HF was designed for a 6-month follow-up which may have determined an underestimation of the number of events, since some scores were originally designed to predict longer time points. Also, this is a retrospective (not pre-specified) analysis of the LUS-HF. Finally, our analysis accounts for a composite endpoint consisting in HF hospitalizations, urgent visits for worsening heart failure and all-cause mortality so it may not be generalized to prognostic risk scores specifically designed for other outcomes.

We consider our study as hypothesis generating and acknowledge the need of testing the hypothesis in other HF larger cohorts, especially multicentric and with a longer follow-up.

## 5 Conclusion

Adding the results of LUS evaluated at discharge improved the predictive value of most of the contemporary HF risk scores. As it is a simple, fast, and non-invasive test it may be recommended to assess prognosis at discharge in HF patients.

## Data Availability

The data that support the findings of this study are available from the corresponding author upon reasonable request.

## References

[B1] Álvarez-García J. Ferrero-Gregori A. Puig T. Vázquez R. Delgado J. Pascual-Figal D. (2015). A simple validated method for predicting the risk of hospitalization for worsening of heart failure in ambulatory patients: The Redin-SCORE. Eur. J. Heart Fail. 17 (8), 818–827. 10.1002/ejhf.287 26011392 PMC5032982

[B2] Araiza-Garaygordobil D. Gopar-Nieto R. Martinez-Amezcua P. Cabello-López A. Alanis-Estrada G. Luna-Herbert A. (2020). A randomized controlled trial of lung ultrasound-guided therapy in heart failure (CLUSTER-HF study). Am. Heart J. 227, 31–39. 10.1016/j.ahj.2020.06.003 32668323

[B3] Aras M. A. Teerlink J. R. (2016). Lung ultrasound: A ‘B-line’ to the prediction of decompensated heart failure. Eur. Heart. J. 37, 1252–1254. 10.1093/eurheartj/ehw094 27080198

[B4] Bettencourt P. Azevedo A. Pimenta J. Friões F. Ferreira S. Ferreira A. (2004). N-terminal-pro-brain natriuretic peptide predicts outcome after hospital discharge in heart failure patients. Circulation 110 (15), 2168–2174. 10.1161/01.CIR.0000144310.04433.BE 15451800

[B5] Codina P. Lupón J. Borrellas A. Spitaleri G. Cediel G. Domingo M. (2021). Head-to-head comparison of contemporary heart failure risk scores. Eur. J. Heart Fail. 23, 2035–2044. 10.1002/ejhf.2352 34558158

[B6] Coiro S. Rossignol P. Ambrosio G. Carluccio E. Alunni G. Murrone A. (2015). Prognostic value of residual pulmonary congestion at discharge assessed by lung ultrasound imaging in heart failure. Eur. J. Heart Fail. 17 (11), 1172–1181. 10.1002/ejhf.344 26417699

[B7] Coiro S. Simonovic D. Deljanin-Ilic M. Duarte K. Carluccio E. Cattadori G. (2020). Prognostic value of dynamic changes in pulmonary congestion during exercise stress echocardiography in heart failure with preserved ejection fraction. Circ Heart Fail junio 13 (6), e006769. 10.1161/CIRCHEARTFAILURE.119.006769 32543975

[B8] Domingo M. Conangla L. Lupón J. de Antonio M. Moliner P. Santiago-Vacas E. (2021). Prognostic value of lung ultrasound in chronic stable ambulatory heart failure patients. Rev. Esp. Cardiol. 74 (10), 862–869. 10.1016/j.rec.2020.07.006 32861606

[B9] Gargani L. Pang P. S. Frassi F. Miglioranza M. H. Dini F. L. Landi P. (2015). Persistent pulmonary congestion before discharge predicts rehospitalization in heart failure: A lung ultrasound study. Cardiovasc. Ultrasound 13 (1), 40. 10.1186/s12947-015-0033-4 26337295 PMC4558829

[B10] Gargani L. Pugliese N. R. Frassi F. Frumento P. Poggianti E. Mazzola M. (2021). Prognostic value of lung ultrasound in patients hospitalized for heart disease irrespective of symptoms and ejection fraction. Esc. Heart Fail. 8 (4), 2660–2669. 10.1002/ehf2.13206 33932105 PMC8318481

[B11] Gustafsson M. Alehagen U. Johansson P. (2015). Imaging congestion with a pocket ultrasound device: Prognostic implications in patients with chronic heart failure. J. Card. Fail. 21 (7), 548–554. 10.1016/j.cardfail.2015.02.004 25725475

[B12] Januzzi J. L. Van Kimmenade R. R. J. Boston P. Utrecht M. (2014). Importance of rigorous evaluation in comparative biomarker studies. J. Am. Coll. Cardiol. 63 (2), 167–169. 10.1016/j.jacc.2013.09.005 24076530

[B13] Khanam S. S. Choi E. Son J. W. Lee J. W. Youn Y. J. Yoon J. (2018). Validation of the MAGGIC (Meta-Analysis Global Group in Chronic Heart Failure) heart failure risk score and the effect of adding natriuretic peptide for predicting mortality after discharge in hospitalized patients with heart failure. PLoS ONE 13 (11), e0206380–13. 10.1371/journal.pone.0206380 30485284 PMC6261415

[B14] Kobayashi M. Gargani L. Palazzuoli A. Ambrosio G. Bayés-Genis A. Lupon J. (2021). Association between right-sided cardiac function and ultrasound based pulmonary congestion on acutely decompensated heart failure: Findings from a pooled analysis of four cohort studies. Clin. Res. Cardiol. 110 (8), 1181–1192. 10.1007/s00392-020-01724-8 32770373

[B15] Levy W. C. Mozaffarian D. Linker D. T. Sutradhar S. C. Anker S. D. Cropp A. B. (2006). The seattle heart failure model: Prediction of survival in heart failure. Circulation 113 (11), 1424–1433. 10.1161/CIRCULATIONAHA.105.584102 16534009

[B16] Lupón J. De Antonio M. Vila J. Peñafiel J. Galán A. Zamora E. (2014). Development of a novel heart failure risk tool: The Barcelona bio-heart failure risk calculator (BCN bio-HF calculator). PLoS ONE 9 (1), e85466. 10.1371/journal.pone.0085466 24454874 PMC3893213

[B17] Lupón J. Simpson J. McMurray J. J. V. de Antonio M. Vila J. Subirana I. (2018). Barcelona bio-HF calculator version 2.0: Incorporation of angiotensin II receptor blocker neprilysin inhibitor (ARNI) and risk for heart failure hospitalization. Eur. J. Heart Fail. 20 (5), 938–940. 10.1002/ejhf.949 28949101

[B18] Marini C. Fragasso G. Italia L. Sisakian H. Tufaro V. Ingallina G. (2020). Lung ultrasound-guided therapy reduces acute decompensation events in chronic heart failure. Heart 106 (24), 1934–1939. 10.1136/heartjnl-2019-316429 32571960

[B19] Mazzola M. Pugliese N. R. Zavagli M. De Biase N. Bandini G. Barbarisi G. (2021). Diagnostic and prognostic value of lung ultrasound B-lines in acute heart failure with concomitant pneumonia. Front. Cardiovasc. Med. 8, 693912. 10.3389/fcvm.2021.693912 34490365 PMC8416771

[B20] Mhanna M. Beran A. Nazir S. Sajdeya O. Srour O. Ayesh H. (2021). Lung ultrasound–guided management to reduce hospitalization in chronic heart failure: A systematic review and meta-analysis. Heart fail. Rev. 1, 821–826. 10.1007/s10741-021-10085-x 33835332

[B21] Pellicori P. Shah P. Cuthbert J. Urbinati A. Zhang J. Kallvikbacka-Bennett A. (2019). Prevalence, pattern and clinical relevance of ultrasound indices of congestion in outpatients with heart failure. Eur. J. Heart Fail. 21 (7), 904–916. 10.1002/ejhf.1383 30666769

[B22] Peterson P. N. Rumsfeld J. S. Liang L. Albert N. M. Hernandez A. F. Peterson E. D. (2010). A validated risk score for in-hospital mortality in patients with heart failure from the American Heart Association get with the guidelines program. Circ. Cardiovasc. Qual. Outcomes 3 (1), 25–32. 10.1161/CIRCOUTCOMES.109.854877 20123668

[B23] Platz E. Jhund P. S. Girerd N. Pivetta E. McMurray J. J. V. Peacock W. F. (2019). Expert consensus document: Reporting checklist for quantification of pulmonary congestion by lung ultrasound in heart failure. Eur. J. Heart Fail. 21 (7), 844–851. 10.1002/ejhf.1499 31218825 PMC6708584

[B24] Platz E. Lewis E. F. Uno H. Peck J. Pivetta E. Merz A. A. (2016). Detection and prognostic value of pulmonary congestion by lung ultrasound in ambulatory heart failure patients. Eur. Heart J. 37 (15), 1244–1251. 10.1093/eurheartj/ehv745 26819225 PMC5006102

[B25] Pocock S. J. Ariti C. A. Mcmurray J. J. V. Maggioni A. Køber L. Squire I. B. (2013). Predicting survival in heart failure: A risk score based on 39 372 patients from 30 studies. Eur. Heart J. 34, 1404–1413. 10.1093/eurheartj/ehs337 23095984

[B26] Pugliese N. R. De Biase N. Gargani L. Mazzola M. Conte L. Fabiani I. (2021). Predicting the transition to and progression of heart failure with preserved ejection fraction: A weighted risk score using bio-humoural, cardiopulmonary, and echocardiographic stress testing. Eur. J. Prev. Cardiol. 28 (15), 1650–1661. 10.1093/eurjpc/zwaa129 33624088

[B27] Rastogi T. Bozec E. Pellicori P. Bayes-Genis A. Coiro S. Domingo M. (2022). Prognostic value and therapeutic utility of lung ultrasound in acute and chronic Heart Failure: A meta-analysis. JACC. Cardiovasc. Imaging 15 (5), 950–952. 10.1016/j.jcmg.2021.11.024 35033496

[B28] Rivas-Lasarte M. Álvarez-García J. Fernández-Martínez J. Maestro A. López-López L. Solé-González E. (2019). Lung ultrasound-guided treatment in ambulatory patients with heart failure: A randomized controlled clinical trial (LUS-HF study). Eur. J. Heart Fail. 21 (12), 1605–1613. 10.1002/ejhf.1604 31667987

[B29] Rivas-Lasarte M. Maestro A. Fernández-Martínez J. López-López L. Solé-González E. Vives-Borrás M. (2020). Prevalence and prognostic impact of subclinical pulmonary congestion at discharge in patients with acute heart failure. Esc. Heart Fail. 7 (5), 2621–2628. 10.1002/ehf2.12842 32633473 PMC7524099

[B30] Scali M. C. Cortigiani L. Simionuc A. Gregori D. Marzilli M. Picano E. (2017). Exercise-induced B-lines identify worse functional and prognostic stage in heart failure patients with depressed left ventricular ejection fraction: Exercise B-lines in heart failure. Eur. J. Heart Fail. 19 (11), 1468–1478. 10.1002/ejhf.776 28198075

[B31] Sinha A. Gupta D. K. Yancy C. W. Shah S. J. Rasmussen-Torvik L. J. McNally E. M. (2021). Risk-based approach for the prediction and prevention of heart failure. Circ Heart Fail, 259–272. 10.3389/fcvm.2021.785109 PMC7887083 33535771

